# Endoscopic Disease Activity in Inflammatory Bowel Disease

**DOI:** 10.1007/s11894-015-0470-0

**Published:** 2015-12-09

**Authors:** Shara Nguyen Ket, Rebecca Palmer, Simon Travis

**Affiliations:** Gastroenterology Unit, John Radcliffe Hospital, Oxford, UK; Translational Gastroenterology Unit, Experimental Medicine Division, Nuffield Department of Medicine, John Radcliffe Hospital, University of Oxford, Oxford, OX3 9DU UK

**Keywords:** Endoscopy, Ulcerative colitis, Crohn’s disease, UCEIS, Mayo score

## Abstract

Endoscopic scoring systems in Crohn’s disease and ulcerative colitis aim to translate the assessment of mucosal disease activity into a quantified value. This value seeks to provide a clear, objective record of the endoscopic mucosal severity, which can then be used to guide medical management decisions. The primary driver of all endoscopic indices, however, is to define a scale of responsiveness for therapeutic endpoints in clinical trials. Mucosal healing now has widespread acceptance as a therapeutic and clinical endpoint, but despite the development of multiple endoscopic scoring systems, the endoscopic definition has yet to be resolved. This review describes recent advances in endoscopic scoring systems for ulcerative colitis (Mayo Clinic endoscopy subscore, UCEIS, and UCCIS among others) and for Crohn’s disease.

## Introduction

Endoscopic scoring systems of disease activity in inflammatory bowel disease (IBD) have a pivotal role in assessment and management. Scoring systems aim to interpret the endoscopic disease appearance and translate this into a quantified score, which introduces standardization and should reduce inter- (and intra-) observer variability. This enables endoscopic endpoints for clinical trials to be defined and facilitates comparisons between trials. A secondary purpose of scoring systems is their use in clinical practice, to guide therapeutic decisions, and to use the scoring system of mucosal appearance as an indicator of the course of the disease or longer term outcome. Mucosal healing is now preferred over clinical remission as a target endpoint [1•], because it has been demonstrated to reduce hospitalization and corticosteroid use, to reduce the risk of colectomy or bowel resection, and to reduce the risk of colorectal cancer [2]. It is also associated with sustained clinical response and improved quality of life [2]. Mucosal healing is not only a treatment target [3, 4••, 5, 6], but can also delete guide adjustment of medical therapy [2]. Based on repeated endoscopic assessment of mucosal disease activity in ulcerative colitis, medical therapy can be adjusted with a treat to target approach [7] and this has been strongly associated with achieving mucosal healing [8•]. The implication of mucosal healing on clinical and therapeutic outcomes has lead to endoscopic appearance being incorporated into primary endpoints in therapeutic trials. It is thus important to have robust, validated scoring systems that enable reliable quantification and interpretation of mucosal appearance.

## Characteristics of Endoscopic Scoring Systems

### The Use of Descriptors

The difficulty in describing the diverse mucosal appearance in IBD has long been recognized. Attempts to develop specific measures of disease activity has led to a plethora of scoring systems being developed for endoscopic disease activity, few of which have undergone formal validation.

From the early stages of developing endoscopic scoring systems, it was noted that discontinuous variables were essential to reduce inter-observer variability [9]. There are, however, numerous proposed descriptors including vascular pattern, mucosal erythema, mucosal granularity, mucosal edema, mucopurulent exudate, bleeding, incidental, and contact friability as well as erosions and ulcers. Mucosal friability has been difficult to define and a source of disagreement between central readers and site investigators in mild to moderate ulcerative colitis (UC) [10••], so newer systems of avoided this descriptor. During the development of the Ulcerative Colitis Endoscopic Index of Severity (UCEIS), the three descriptors of vascular pattern, bleeding and erosions, or ulcers, were found to account for 86 % of the variability in evaluation of overall severity by visual analogue scale (VAS), which has provided simple, pre-defined, and tested terms for describing the mucosal appearance in ulcerative colitis.

### Ease of Use

An endoscopic scoring system that is to be widely used both in clinical trials and clinical practice needs to be simple to use. All indices require training to be used to best effect. The Crohn’s Disease Endoscopic Index of Severity (CDEIS), for example, although widely used in multiple clinical studies and randomized controlled trials is complex, requires experience, and is not commonly used in general endoscopic practice. The CDEIS total score ranges from 0 to 44. The Simple Endoscopic Score for Crohn’s Disease (SES-CD) was developed in response to the complexities of the CDEIS and uses four descriptors each allocated to the five bowel segments.

### Validation and Adequate Observer Variability

The strength of intra-observer or inter-observer agreement is commonly evaluated according to the criteria of Landis and Koch, whereby interclass correlation coefficients of <0.00, 0.00 to 0.20, 0.21 to 0.40, 0.41 to 0.60, 0.61 to 0.80, and 0.81 to 1.00 represent poor, slight, fair, moderate, substantial, and almost perfect agreement, respectively [11].

A study (2014) has assessed the reproducibility of four endoscopic scoring systems: Mayo subscore for ulcerative colitis, Rutgeerts score for postoperative Crohn’s disease, CDEIS, and SES-CD. Fourteen gastroenterologists experienced in endoscopic scoring for IBD and 30 gastroenterologists who had not received specific training in endoscopic scores reviewed 31 endoscopic videos of IBD. The Mayo subscore demonstrated suboptimal agreement with a *κ* score of 0.53 and 0.71 for the two groups. The Rutgeerts *κ* score for experienced and inexperienced groups were 0.57 and 0.67. Intra-class correlation coefficients (ICC) for the CDEIS were 0.83 and 0.67, and for SES-CD were 0.93 and 0.68 [12], respectively. This inconsistency among gastroenterologists in assessing endoscopic disease severity can result in variations in rates of response in therapeutic IBD trials that can affect licensing [10••]. In a study of mesalamine in mild to moderately active UC, there was substantial disagreement between site readers and central reader, with an ICC of 0.11 at screening. This resulted in 31 % of patients enrolled being considered ineligible by a central reader. Exclusion of these patients substantially reduced the remission rate in the placebo group [10••]. As a consequence of central reading, the clinical trial outcome changed from one lacking significance to one that was significant. In another study, central reading reduced overall variability of scores, particularly in mild to moderate endoscopic disease severity, represented by a UCEIS score of between 3 and 5 [13]. These studies highlight two principles:

(i) Central reading in trials reduces variation in endoscopic interpretation, and
(ii) The need to select a validated endoscopic scoring system that has limited inter-observer variability.

These factors potentially influence the outcome of clinical trials.

A further study (2014) assessed whether commonly used disease activity indices for UC were reliable and clinically relevant. One hundred consecutive patients with ulcerative colitis presenting for review had clinical symptoms recorded and a video sigmoidoscopy performed on the same day. The Simple Clinical Colitis Activity, Mayo Clinic index, and Seo indices were compared with an inter-observer agreement of between *κ* 0.72 and 0.89 for the three indices; however, the Mayo Clinic index had the greatest variation (*κ* = 0.38) [14].

The UCEIS is the first validated tool for assessing endoscopic disease activity in ulcerative colitis and scores the worst affected area at flexible sigmoidoscopy. Validation was performed in a study where 25 investigators blinded to clinical information assessed 28 videos using the descriptors from the UCEIS and a VAS to assess overall severity. There was high intra-investigator and inter-investigator reliability ratios of 0.96 and 0.88, respectively [15••].

## Current Updates in Scoring Indices in Ulcerative Colitis

Clinical implications of endoscopic scoring systems are important to enable the meaningful application of the scores. The impact of the knowledge of clinical information on endoscopic scores has been studied (2015). In a study using a cohort of independent central reader investigators, the knowledge or absence of clinical information was found to have minimal impact on mean UCEIS scores of sigmoidoscopies. Intra-reader *κ* scores for the full UCEIS were 0.51 and 0.56 for the blinded and unblinded readers, respectively, which was not significantly different (*p* = 0.66) [13]. This study also found the UCEIS to correlate well with contemporaneously recorded symptoms. The Modified Baron Score correlated less well, but no comparison could be made with the Mayo Clinic index because patient-reported symptoms were derived from subscores of that index [13]. The responsiveness of the Modified Mayo Clinic Endoscopic Score, Modified Baron Score, and UCEIS to clinical change have also been studied in a randomized placebo-controlled trial of mesalamine therapy. All three indices displayed similar responsiveness for changes of UC disease activity, but the UCEIS was numerically better in all regards and the larger scale (0–8 compared to 0–3 in the Mayo Clinic endoscopy subscore) may have advantages in the evaluating response [16]. This has proved to be the case in a Japanese study (2015) comparing the Mayo Clinic and UCEIS endoscopy scores for detecting response to treatment with tacrolimus: the UCEIS detected change over an interval of 3 months, while the Mayo clinic score did not [17].

In acute severe colitis, the UCEIS helps predict outcomes. A study of 89 consecutive causes of acute severe colitis admitted to a single institution were retrospectively reviewed, and admission endoscopic disease severity by UCEIS found to correlate with the outcome. The study suggested a strong likelihood of needing rescue therapy with ciclosporin or infliximab for a high UCEIS score; 11/14 (79 %) with a UCEIS score ≥7 required rescue therapy. There was a significant association between UCEIS and colectomy, when the UCEIS was ≥5, 33 % required colectomy compared to 9 % ≤4 (*p* = 0.037) [18•].

Current scores for ulcerative colitis do not however, take into account extent and distribution of mucosal inflammation, and a Modified Mayo Endoscopic Score (MMES) has been developed to address this (2015). Dividing the colon into five segments, the Mayo endoscopic subscore for each segment is added together to give a Modified Score. This Modified Score is then multiplied by the maximal extent of inflammation then divided by the number of segments with active inflammation to give the MMES. The MMES correlates with clinical, biological, and histological activity, but has yet to be validated [19•]. Extent, however, is another dimension to endoscopic severity. It is relevant to assessing the overall severity of UC, but so are systemic features (pulse rate, temperature, anemia) and treatment responsiveness. For this reason, our belief is that extent is best rated separately and not mixed with assessment of endoscopic mucosal severity. This avoids adding the complexity of segmental assessment to the endoscopic evaluation of UC. Beware the CDEIS! The UCEIS evaluates the worst-affected mucosa at flexible sigmoidoscopy [20].

Yet another dimension integral to endoscopy is the histological assessment of disease severity in mucosal biopsies. Although historically endoscopically active UC does not correlate well to histological severity, until now there have been no validated scores to compare. Histological remission in UC may yet have predictive value. In a post hoc analysis comparing endoscopic assessment using the Rachmilewitz index and histological assessment, 52/380 (14 %) reported endoscopically active UC but *no* histological signs of active inflammation at baseline. Of these patients, 48/52 (92 %) reached clinical remission in a clinical trial, but it can be argued that the absence of histological disease activity meant that they were in remission in the first place [21]. This disparity matters to the recruitment of patients to clinical trials testing therapy for active UC. In contrast, histological remission predicted outcome over a 6-year follow-up period with regard to corticosteroid use and hospitalization with acute severe colitis, while endoscopic mucosal healing did not [22••]. Histologic endpoints may have a role as a surrogate marker of long-term outcomes [23].

## Current Updates in Scoring Indices in Crohn’s Disease

The precision of the Rutgeerts score has recently been called into question, although it has been used in clinical practice and therapeutic trials since 1990. However, the Rutgeerts score has not been prospectively validated and the definition of postoperative recurrence, commonly defined as a score of ≥i2, has been questioned. In the POCER trial (2015), defining postoperative recurrence as a Rutgeerts score ≥i3 would have resulted in a non-statistically significant difference between active care and standard care arms [22••]. In another prospective, multicenter trial which included patients with Crohn’s disease higher risk of recurrence after ileocolic resection, patients were randomized to systematic azathioprine ≤2 weeks from surgery, or endoscopy-driven initiation of azathioprine for patients with a Rutgeerts score ≥i2 at week 26 or 52 following surgery. No significant difference was demonstrated in the primary endpoint of endoscopic remission (Rutgeerts score ≥i0-i1) at week 102 between the two groups, although the study was prematurely stopped due to slow recruitment [24•]. A third prospective study (PREVENT) of infliximab versus placebo for patients at high risk of recurrence after ileocolic resection and anastomosis was stopped prematurely, because the event rate (clinical relapse) was lower than predicted by endoscopy, although the results have yet to be published. This calls into question the value of the Rutgeerts score for clinical decision-making, particularly the i2 subscore which groups lesions at the anastomosis and neoterminal ileum together.

In an attempt to determine a definition for endoscopic response and predict sustained clinical benefit, the SES-CD and CDEIS scores from the SONIC trial were. After central readers calculated scores at baseline and a subsequent endoscopy after 26 weeks’ treatment with infliximab, azathioprine or both, *endoscopic response* was defined as a ≥50 % decrease from baseline in SES-CD or CDEIS. Endoscopic response at 26 weeks, according to this definition, was predictive of corticosteroid-free clinical remission at week 50 [5]. Although this proposed cutoff appears to be a relevant endpoint, further validation and evaluation are required.

## Conclusion

Endoscopic scoring systems are necessary for standardized reporting of mucosal appearance in inflammatory bowel disease, both in clinical trials and practice. The UCEIS is emerging as a useful index with clinical and therapeutic relevance that has been validated with good intra- and inter-observer variability. How the UCEIS and Mayo Clinic endoscopic score compare needs further study in prospective therapeutic trials that are currently in progress, but data to date suggest an advantage for the UCEIS. The Rutgeerts postoperative endoscopic score, although widely used, needs to be re-examined, particularly the definition of the subscore of i2. Simplification of endoscopic scoring systems for CD would increase their value if the key components that predict outcomes (such as a score at the most diseased area) could be defined. The clinical relevance of endoscopic response to treatment, endoscopic remission, and mucosal healing will remain elusive until there is general accord on their definition.
